# Identification of phylogenetically conserved sequence motifs in microRNA 5' flanking sites from *C. elegans *and *C. briggsae*

**DOI:** 10.1186/1471-2199-9-105

**Published:** 2008-11-26

**Authors:** Liisa Heikkinen, Suvi Asikainen, Garry Wong

**Affiliations:** 1 Department of Biosciences, University of Kuopio, PO Box 1627, Kuopio 70211, Finland; 2 Department of Neurobiology, AI Virtanen Institute, University of Kuopio, PO Box 1627, Kuopio 70211, Finland

## Abstract

**Background:**

MicroRNAs (miRNAs) are small, noncoding RNA molecules that act as post-transcriptional regulators of gene expression. Studies concerning transcriptional regulation of miRNAs have so far concentrated on those located within the intergenic region of the genome and the search for putative promoters, thus leaving open the question of the existence of possible regulatory elements common to all miRNAs including those located in introns of protein coding genes.

**Results:**

In this study, we initially searched for motifs occurring in the area 1000 bp upstream from all miRNAs independent of their genomic location. We discovered a previously unknown sequence motif GANNNNGA that displayed a conserved distribution in the nematode worms *Caenorhabditis elegans *and *Caenorhabditis briggsae*. This motif had a peak occurrence at 500 bp upstream, with a sharp drop-off toward the miRNA start site. Further analysis indicated that this motif was locally restricted and not enriched 1000–5000 bp upstream or 0–2000 bp downstream of the miRNA start site. In addition, this motif was observed to be most abundant in the upstream sequences of two important miRNAs, *mir-1 *and *mir-124*. This abundance was also conserved in phylogenetically distant species including human and mouse.

**Conclusion:**

The results show that the motif GANNNNGA is conserved close to miRNA precursor start sites, suggesting that it may be involved in miRNA sequence recognition or regulation. This data provides important knowledge for the identification and computational prediction of miRNA sequences.

## Background

MicroRNAs (miRNAs) form one class of small, non-protein coding RNAs. They are defined as single stranded, 19–25 nt long endogenously expressed RNAs generated from one arm of a hairpin precursor sequence [1-3]. These precursors are derived from a larger transcript, pri-miRNA, which is considered to be the original transcription product of a miRNA gene. MiRNAs are known to be involved in the post-transcriptional regulation of protein-coding genes. They operate by binding to the 3'UTR region of a mRNA sequence with antisense base pairing and cleave the target mRNA or repress its translation into protein. One miRNA can repress various different mRNAs and a single mRNA may be bound by several co-operating miRNAs [4,5]. The expression of a miRNA may be associated with different developmental stages of an organism or, more often, they are found to be expressed in particular cell types [2,3].

The transcriptional regulation of miRNA genes is still not completely understood. Most miRNA genes are located in the genome far away from protein coding genes and are therefore thought to derive from their own, independent transcription units [2,3]. There is also some evidence that these "intergenic" miRNAs are transcribed from their own promoters by RNA Polymerase II [6,7], and that a small fraction of miRNAs interspersed among the Alu repeats in human genome may be transcribed by RNA Polymerase III [8]. MiRNAs organized to clusters are also suggested to be transcribed independently as multi-cistronic units [9,10]. Those miRNAs whose origins are in introns of protein coding genes are possibly transcribed either in parallel with their host genes or independently [2,3].

The upstream regions of intergenic miRNAs have been inspected in several studies to reveal additional information about miRNA transcription [7,11-13]. In these studies, some interesting sequence motifs have been found and also a few known transcription factors are suggested to regulate the miRNAs. The focus has thus far mainly been in the putative promoters of miRNA genes that are located in the intergenic area, thus leaving open the question of possible regulatory elements of all the other miRNAs.

In this study, we examined the upstream sequences of *Caenorhabditis elegans *and *Caenorhabditis briggsae *miRNAs in order to find over-represented, phylogenetically conserved short sequence motifs that occur upstream from miRNAs, independent of their origin in the genome. In the analysis, we used four motif finding tools with different algorithms and motif models. The most significant motif found was GANNNNGA, a novel motif with conserved distribution upstream of the miRNAs in these worms. This motif was also found to be especially abundant in the upstream sequences of two old and biologically important miRNAs, *mir-1 *and *mir-124*, thus suggesting a connection between the number of motif instances in the upstream sequence close to a miRNA start site and a globally conserved function of the miRNA.

## Results

**Figure 1:**
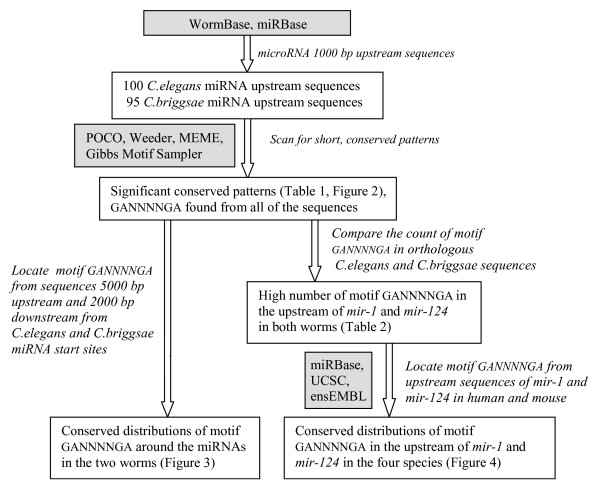
The main workflow of this study.

The main workflow of the study is shown in Figure 1.

### Search for conserved motifs

The putative miRNA promoter sequence sets, containing the sequences up to 1000 bp upstream from the starting site of 100 *Caenorhabditis elegans *miRNAs (25 of these were located within a protein coding gene) and 95 *Caenorhabditis briggsae *miRNAs, were searched for conserved over-represented sequence patterns with four motif finding tools: POCO, Weeder, MEME and Gibbs Motif Sampler.

With POCO, 65 significant patterns that occur in every sequence in both *C. elegans *and *C. briggsae *putative miRNA promoter sequence sets were found (Table 1). The found patterns were 4–8 bp long, containing four known nucleotides A, C, G or T while the rest of the places in a pattern, marked as N, can be any of these. There were four patterns having an average of more than 10 occurrences in both of our putative miRNA regulatory region data sets: GAGA (11.6 in *C. elegans*/15.4 in *C. briggsae*), GANNNNGA (11.3/14.8), GANANNG (11.0/13.1) and ANAGNG (10.9/11.0), thus containing abundant A and G. To check if these nucleotides are dominating in our putative regulatory sequences, the proportion of all nucleotides was calculated from all the sequences of *C. elegans *and *C. briggsae*. The most frequent nucleotides were A (30.9% in *C. elegans *and 29.9% in *C. briggsae*) and T (30.8% and 29.7%, respectively), while both C and G had about 20% proportions. The multiple sequence alignment of the over-represented patterns common to both data sets gave for consensus sequence GANNNNGNG, a pattern that closely resembles GANNNNGA, one of the most copious patterns found from both putative miRNA regulatory sequence sets. Thus, from all the POCO results, we selected GANNNNGA for further study. In addition, to find if this actually was a repetitive motif, we counted also the occurrences of GANNNNGANNNNGA and found it from 57 *C. elegans *and 58 *C. briggsae *putative miRNA regulatory sequences.

**Table 1:** Significant patterns found with POCO from 1000 bp upstream sequences of *C. elegans *and *C. briggsae*

Index	Pattern	C. elegans	C. briggsae	Index	Pattern	C. elegans	C. briggsae
**1**	GAGA	11.61	15.38	**34**	ANAGNNNC	6.76	7.64
**2**	GANNNNGA	11.29	14.81	**35**	GGNNGA	6.63	8.29
**3**	GANANNG	10.95	13.06	**36**	GANGNG	6.62	8.61
**4**	ANAGNG	10.93	10.99	**37**	GNANNNGG	6.62	8.25
**5**	ANNNGNAG	9.74	11.04	**38**	GNANGNG	6.27	7.42
**6**	GANNNAG	9.70	11.64	**39**	GNAGNNNG	6.25	8.05
**7**	AGNGNNNA	9.50	9.56	**40**	GNNGNGNA	5.99	8.04
**8**	ANGNNGA	9.49	11.14	**41**	AGNGNNC	5.92	5.69
**9**	AGNGNA	9.40	9.60	**42**	GGNGG	5.91	7.64
**10**	GAGNG	9.36	9.48	**43**	GNNANNGG	5.91	6.96
**11**	AGNGA	9.35	11.93	**44**	ANNGGNNG	5.79	7.29
**12**	ANNGNNGA	9.01	10.00	**45**	GNGNNGG	5.73	7.65
**13**	GAANG	8.85	11.34	**46**	GNGNANG	5.73	6.83
**14**	AGNAG	8.75	11.58	**47**	ANGNGG	5.72	7.46
**15**	ANANGNG	8.65	10.35	**48**	ANNGGNG	5.59	6.84
**16**	GNNNANGA	8.53	11.19	**49**	ANNGNGG	5.56	6.56
**17**	AGNGNG	8.44	8.23	**50**	GGNNNANG	5.48	7.39
**18**	ANNNAGNG	8.33	10.28	**51**	ANNNGGC	5.45	5.79
**19**	GANGNNG	8.17	10.05	**52**	ANGNGNG	5.36	7.00
**20**	GNGNGNG	8.07	9.44	**53**	AGGNG	5.34	7.09
**21**	GNNGNNGA	7.91	9.69	**54**	GGNNNNGG	5.28	7.55
**22**	ANGAG	7.79	10.74	**55**	GNGNNANG	5.27	6.83
**23**	ANANGG	7.78	9.83	**56**	ANGGNG	5.26	6.61
**24**	AAGNG	7.75	10.14	**57**	AGNGG	5.21	6.80
**25**	GANNGNG	7.65	8.82	**58**	GGNNAG	5.07	6.65
**26**	GNNNGAG	7.59	9.32	**59**	GTNGNNNG	5.00	5.58
**27**	GNGAG	7.58	9.06	**60**	GGNNNNAG	4.92	6.89
**28**	AGNNNGNG	7.52	8.58	**61**	ANGGNNNG	4.89	6.69
**29**	GNNGAG	7.41	9.65	**62**	CGANNG	4.86	5.27
**30**	AGNANNG	7.41	9.22	**63**	ANGNNNCG	4.42	5.47
**31**	ANNGNGNG	7.17	8.95	**64**	GGNNNGG	4.38	7.20
**32**	GNNGNGA	6.92	8.44	**65**	ANNNGGNC	4.26	5.18
**33**	AGNNNAG	6.80	8.61				

The patterns are listed in the order of their average occurrences.

**Figure 2:**
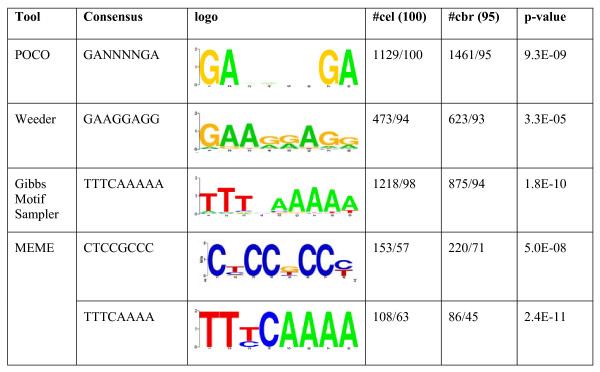
**Significant conserved motifs discovered from the miRNA regulatory region of *C. elegans *and *C. briggsae***. Presented is the total number of the motif instances in both regulatory region sequence sets and the number of miRNAs that contain the motif in their upstream region. The rightmost column shows the *p*-value of the motif calculated against random background sequences. Motif logos show the information content present at every position in the sequence, for example in the logo of motif GANNNNGA the G:s and A:s are certain, thus having the highest information content (2 bits) while in the positions 3 to 6 all four nucleotides have about the same frequencies and the information content in these positions is close to zero.

Both putative miRNA regulatory sequence sets were further scanned for over-represented short patterns using three motif finding tools with different algorithms and motif models. With Weeder, the most significant pattern found was GAAGGAGG, located 473 times from 94 *C. elegans *and 623 times from 93 *C. briggsae *miRNA upstream sequences. Further, with Gibbs Motif Sampler, the most frequent motif revealed was TTTCAAAAA, with 1218 instances in 98 *C. elegans *sequences, but only 623 instances in 94 *C. briggsae *sequences. The two most significant patterns found with MEME had consensus sequences CTCCGCCC and TTTCAAAA, the latter of which is a more well-defined version of the motif with similar consensus found with Gibbs Motif Sampler. These two motifs were found only from 57 and 63 *C. elegans *miRNA upstream sequences, respectively. All the motifs found with different tools together with the motif sequence logos and the motif *p*-values calculated against random background are gathered to Figure 2.

### Motif analysis

The Transfac 7.0 public database [14] contains only a few nematode transcription factor binding sites, and none of them could be matched with any motif we had found.

We did find a human match with ISGF-3 from Transfac, and STAT1 from the JASPAR v3 database [15]. Some of these motifs we observed in *C. elegans *were reported in earlier studies concerning *C. elegans *intergenic miRNA promoter elements. The motif CTCCGCCC was first found by Ohler et al. [11], from conserved upstream sequence blocks of orthologous, independently transcribed *C. elegans *and *C. briggsae *miRNA foldbacks and later, this same motif was found also by Zhou et al. [7]. Also, the motif TTTCAAAAA was found earlier by Ohler et al. [11] from the area 1000 bp downstream of orthologous, independently transcribed *C. elegans *and *C. briggsae *miRNA foldbacks. We found this motif upstream in almost every *C. elegans *miRNA, including 16 miRNAs from the sense strand of an intron. This may not be surprising, because a motif very similar to this was discovered earlier from *C. elegans *protein coding gene introns [16]. Most miRNA genes have CT-repeats in their nearby upstream region, which may suggest significance of the motif GAAGGAGG in the studied area [7]. In contrast, the motif GANNNNGA has equal frequencies of different nucleotides in positions 3 to 6, and any link to the CT-repeat is not, at this stage, obvious.

### Conservation studies of the motif GANNNNGA

**Figure 3:**
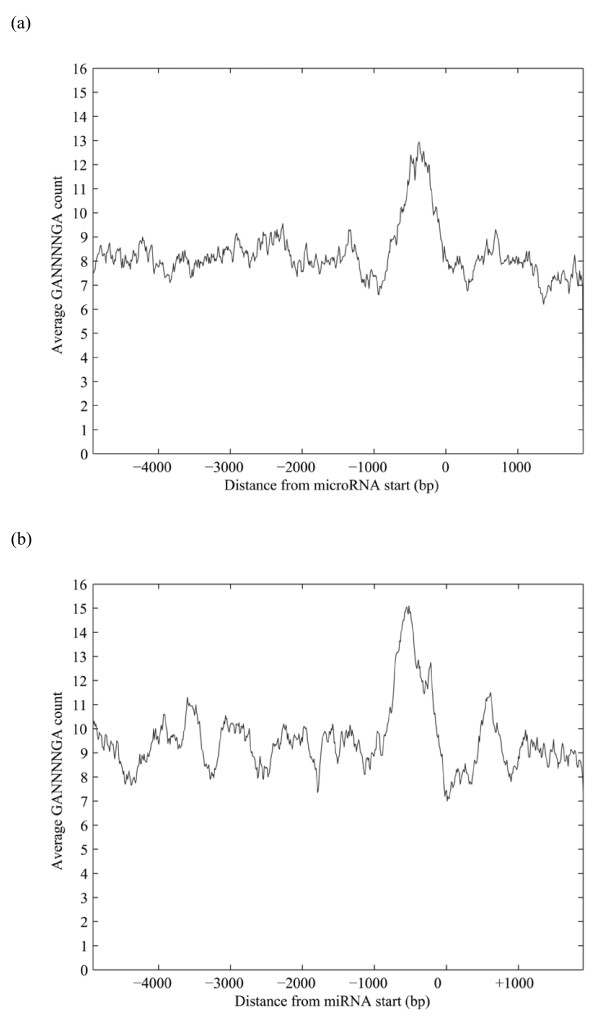
**The frequency distribution of motif GANNNNGA around the miRNAs of *C. elegans *(a) and *C. briggsae *(b)**. All occurrences of the motif GANNNNGA were located from the sequences 0–5000 bp upstream and 0–2000 bp downstream of all *C. elegans *and *C. briggsae *miRNA start sites and the corresponding 200 bp central moving average curves were drawn for both worms.

GANNNNGA was the only motif that occurred in all studied putative miRNA promoter sequences in both species. To determine whether this motif is specific to the studied area up to 1000 bp upstream from the starting site of nematode miRNAs, we downloaded the sequences from 5000 upstream to 2000 bp downstream with respect to miRNA start sites for all miRNAs of both worms, and located all the motif occurrences from this area. Further, we drew the 200 bp moving average curves of the frequency of motif GANNNNGA for both worms in this larger area around the miRNAs (Figure 3). The curves show that the motif GANNNNGA has conserved distribution relative to miRNA starting sites in both worms, and that it is most frequent in the area 1000 bp upstream from the miRNA start site with a pronounced peak at about 500 bp with ~13 occurrences on average in *C. elegans *and ~15 occurrences in *C. briggsae*, while the overall average count of the motif is less than 10 in both worms.

All occurrences of the motif GANNNNGA were then counted from 1000 bp upstream of 70 orthologous miRNA pairs in *C. elegans *and *C. briggsae*. The results of this calculation and the location of each miRNA with respect to the *C. elegans *protein coding genes are presented in Table 2. For those miRNAs that are from the protein coding area, we also indicate its strand with respect to the host gene (sense +, antisense -). Fifteen orthologous miRNA pairs have 15 or more instances of the motif GANNNNGA in the area 1000 bp upstream from their start sites, including 7 miRNAs from the sense strand of an intron and 1 miRNA overlapping an exonic area. On the other hand, 34 of the 70 orthologous miRNAs have less than 10 motif GANNNNGA occurrences in *C. elegans*. When comparing these results, we found that the motif GANNNNGA is especially abundant in the 1000 bp upstream sequences of miRNAs *mir-1*, *mir-124 *and *mir-228 *in both worms. From these, *mir-1 *and *mir-228 *are intergenic, while *mir-124 *is located in the intron of the gene *trpa-1*.

**Table 2:** Motif count of GANNNNGA in the 1000 bp upstream sequences of orthologous miRNAs of *C. elegans *and *C. briggsae*

**Index**	**miRNA**	**CEL gene relationship**	**CEL**	**CBR**	**Index**	**miRNA**	**CEL gene relationship**	**CEL**	**CBR**
**1**	mir-228	intergenic	52	29	**36**	mir-268	Intergenic	10	23
**2**	mir-47	Intergenic	27	29	**37**	mir-70	Intronic +	9	9
**3**	mir-233	Intronic +	26	16	**38**	mir-253	Intronic +	9	10
**4**	mir-124	Intronic +	26	30	**39**	mir-353	Intronic +	9	10
**5**	mir-239.2	Intergenic	24	13	**40**	mir-359	Intergenic	9	10
**6**	mir-241	Intergenic	23	10	**41**	mir-42	Intergenic	9	19
**7**	mir-52	Intergenic	23	25	**42**	mir-254	Intronic +	8	6
**8**	mir-1	Intergenic	23	26	**43**	mir-240	Intergenic	8	7
**9**	mir-239.1	Intergenic	22	13	**44**	mir-45	Intergenic	8	10
**10**	mir-87	Intronic +	18	15	**45**	mir-57	Intergenic	8	18
**11**	mir-62	exon overlap	18	21	**46**	mir-79	Intergenic	8	34
**12**	mir-71	Intronic +	18	34	**47**	mir-360	Intergenic	7	8
**13**	mir-58	Intronic +	17	16	**48**	mir-235	Intergenic	7	9
**14**	mir-82	Intronic +	17	22	**49**	mir-248	Intergenic	7	10
**15**	lin-4	Intergenic	17	23	**50**	mir-232	Intergenic	7	11
**16**	mir-86	Intronic -	17	24	**51**	mir-34	Intergenic	7	12
**17**	mir-49	Intergenic	16	10	**52**	mir-242	Intergenic	7	14
**18**	mir-85	intronic +	16	13	**53**	mir-81	Intronic -	7	14
**19**	mir-354	3' UTR +	16	14	**54**	mir-245	Intergenic	7	15
**20**	mir-90	Intergenic	16	27	**55**	mir-231	Intergenic	6	10
**21**	mir-77	Intergenic	15	8	**56**	mir-60	Intergenic	6	12
**22**	mir-35	intronic -	15	11	**57**	mir-51	Intergenic	6	18
**23**	mir-244	Intergenic	15	16	**58**	mir-83	Intergenic	6	18
**24**	mir-84	Intergenic	14	18	**59**	mir-61	Intergenic	6	21
**25**	mir-251	Intergenic	13	9	**60**	mir-392	Intergenic	5	5
**26**	mir-80	Intergenic	13	15	**61**	mir-234	Intergenic	5	9
**27**	mir-73	Intergenic	13	16	**62**	mir-356	3' UTR +	5	9
**28**	mir-50	Intronic +	12	11	**63**	mir-355	Intergenic	5	10
**29**	mir-259	Intergenic	12	13	**64**	mir-358	Intronic -	5	10
**30**	mir-67	Intronic +	12	18	**65**	mir-236	Intergenic	5	11
**31**	mir-46	Intergenic	11	19	**66**	mir-72	Intergenic	5	14
**32**	let-7	Intergenic	10	5	**67**	mir-230	Intergenic	5	15
**33**	mir-255	Intergenic	10	9	**68**	lsy-6	Intergenic	5	18
**34**	mir-246	Intergenic	10	10	**69**	mir-75	Intergenic	4	14
**35**	mir-252	Intergenic	10	16	**70**	mir-249	Intergenic	3	12

Included in the table is the location of each miRNA with respect to *C. elegans *protein coding genes and the strand of the miRNAs in the protein coding area relative to their host gene. CEL gene relationship indicates the genomic location. "+" or "-" indicates the strand location. CEL, *C. elegans*; CBR, *C. briggsae*.

To determine whether the motif GANNNGA is conserved in the upstream sequences of *mir-1*, *mir-124 *and *mir-228 *orthologs in other species, we located all its occurrences from 1000 bp upstream sequences of all miRNAs that, according to miRBase [17], belong to *mir-1 *or *mir-124 *family in human and mouse genomes. *Mir-228 *is not found from these two genomes, and generally it is included in the *mir-124 *family [18]. Both human and mouse, have three miRNAs belonging to the *mir-1 *-family: *mir-1-1, mir-1-2 *and *mir-206*. The 1000 bp upstream sequence of human *hsa-mir-1-1 *includes 21 occurrences of the motif which is more than twice as many occurrences as the corresponding sequences of *hsa-mir-1-2 *and *hsa-mir-206 *which contain 10 and 6 occurrences, respectively. For mouse, the numbers are 20 for *mmu-mir-1-1*, 6 for *mmu-mir-1-2*, and 6 for *mmu-mir-206*. Similar results were observed when looking at the occurrences of GANNNNGA in the *mir-124 *-family, where the putative promoter sequence of *mir-124a-1 *is the only one that includes a significant number of the motif in both species, 37 and 19, respectively. In summary, the *mir-1-1 *1000 bp upstream sequences of human and mouse contain nearly as many occurrences of the motif GANNNNGA (21 and 20) as the corresponding sequences of *mir-1 *of *C. elegans *and *C. briggsae *(23 and 26). The same holds for the 1000 bp upstream sequences of *mir-124-a1 *of human and mouse (37 and 19) compared with the corresponding sequences for *mir-124 *of *C. elegans *and *C. briggsae *(26 and 30). On average, the human miRNA 1000 bp upstream sequences contain 9.9 occurrences of motif GANNNNGA and in mouse the corresponding average is 10.6 compared to 11.3 and 14.8 for *C. elegans *and *C. briggsae*, respectively.

**Figure 4:**
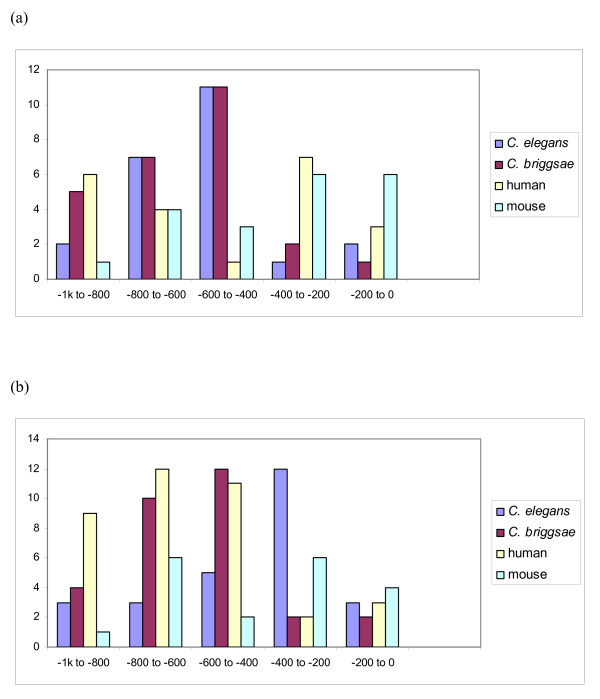
**The frequency diagrams of the motif GANNNNGA occurrences in sequences 1000 bp upstream of *mir-1 *(panel a) and *mir-124 *(panel b) family members in *C. elegans*, *C. briggsae*, human and mouse**. The *mir-1-1 *and *mir-124-a1 *1000 bp upstream sequences of human and mouse contain similar number of occurrences of the motif GANNNNGA as the corresponding sequences in *C. elegans *and *C. briggsae*.

We draw the frequency diagrams of the motif GANNNNGA in the 1000 bp upstream sequences of *mir-1 *and *mir-124 *orthologs in these four species (Figure 4), and made the global alignments of *mir-1 *and *mir-124 *upstream sequences (Additional files 1 and 2). In both cases, we chose *mir-1-1 *as the representative of *mir-1 *-family and *mir-124a-1 *for the representative of *mir-124 *-family for human and mouse. The distribution of motif GANNNNGA in the 1000 bp upstream area of both miRNAs looks rather similar, giving a clue to possible conservation of the motif even between these phylogenetically distant species.

**Figure 5:**
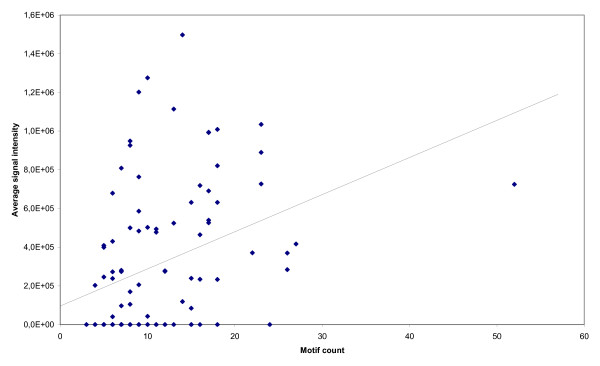
**miRNA microarray expression analysis in *C. elegans***. Dots represent the absolute expression level (vertical axis) of wildtype N2 *C. elegans *miRNAs from whole L4 stage animals as a function of the number of GANNNNGA motifs (horizontal axis). The line indicates a regression plot of the data (r^2 ^= 0.13).

Using miRNA microarray analysis, we measured the signal intensity of *C. elegans *miRNAs isolated from age synchronized whole body wildtype worms and plotted the values versus the number of observed GANNNNGA motifs for each miRNA (Figure 5). A weak correlation only was found, r^2 ^= 0.13.

## Discussion

In this study, we examined the 1000 bp upstream regions of all C. *elegans *and C. *briggsae *miRNAs to reveal characteristic sequence motifs. In addition to the miRNAs in the intergenic area, we included in our study also the miRNAs that lie in the protein coding area and whose possible promoters were not studied earlier. In the search, we focused on over-represented, phylogenetically conserved short sequence motifs that can be found from the miRNA upstream regions, independently of miRNA origins in the genome. The four motif finding algorithms used in the search gave a couple of very frequent result patterns whose consensus sequences were quite diverse, due to the different motif and background models used in the algorithms.

We found one significant sequence motif, GANNNNGA, which was quite abundant and could be located from all miRNA upstream sequences of *C. elegans *and *C. briggsae*. Thus, the motif GANNNNGA is found from all studied miRNA upstream regions, and no clear difference can be made in its abundance when comparing the intergenic miRNAs and the miRNAs located in the protein coding area. In addition, this pattern seemed to also have a conserved distribution in the area surrounding the studied 1000 bp miRNA upstream loci in these two worms, having most of its occurrences between 0–1000 bp upstream from miRNA start sites. From earlier studies [11], this is known to be a highly conserved region upstream of intergenic *C. elegans *and *C. briggsae *orthologous miRNA precursors, and the putative PolII type promoter sequences of most *C. elegans *intergenic miRNAs are predicted to be situated on the 0–500 bp area upstream from the precursors [7]. The motif GANNNNGA was not found in earlier studies of *C. elegans *miRNA promoters [7,11] perhaps because these studies were focused on the intergenic miRNA promoters, thus leaving motifs in the coding area out of scope. Another reason is the tools used for motif finding had a motif model different from that used in POCO, which puts the wildcards (N) in certain places in the middle of the motif and keeps the other places occupied with constant nucleotides [19].

Several lines of evidence suggest that the motif GANNNNGA is not a miRNA promoter. First, although the motif is found proximal to the miRNA transcript, it is found in abundant copies, which is more characteristic of enhancer or other regulatory elements. Second, we have cloned the 1000 bp 5' flanking region of *mir-1 *and used it to construct an expression plasmid with green fluorescent protein as the reporter (data not shown). We were not able to express the reporter despite successful experiments with a variety of other promoters [20]. Eventually, large scale deletion analysis of multiple miRNA flanking sequences using a strong promoter and including the miRNA sequence may be necessary to help define the context where GANNNNGA is active. Thirdly, there appears to be only a weak correlation between the number of GANNNNGA motifs and absolute expression levels (Figure 5). Finally, using STAMP [21], we were not able to find any matching transcription factor binding sites in *C. elegans *using the TRANSFAC or JASPAR databases.

Prior to and independent from the current study, there has been keen interest in *mir-1 *and *mir-124*, two miRNAs with the most abundant GANNNNGA motifs. These two miRNAs are found from all bilateria and are thus interpreted to be among those most phylogenetically ancient miRNAs [22,23]. Both are known to have high tissue specificity. *Mir-1 *is known to have high expression levels in mammalian heart and muscle cells while *mir-124 *contributes to the cells of the nervous system [24]. In a landmark study, Lim et al. [5] demonstrated through transfection to human HeLa cells, that these miRNAs can downregulate the transcription of 96 and 174 annotated genes, respectively, most likely through 3' UTR seed matches, and thus may have a wide global effect to the identity of the tissues mentioned above [5]. The down regulation of brain mRNAs by *mir-124 *in transfected HeLa cells suggests that this miRNA confers tissue specific expression. Consistent with this result, the transfection of *mir-1 *duplexes to HeLa cells produced a heart and skeletal muscle profile. *Mir-124 *has also been shown to promote neuronal differentiation by down regulating the general splice regulator PTB1 [25]. From the evolutionary point of view, the tight tissue specific regulation and large number of genes targeted appears logical, since phylogenetically ancient miRNAs are supposed to have more targets than more recent ones [22]. Consistent with these studies, we found that another brain specific miRNA, *hsa-mir-9*, has 21 occurrences of motif GANNNNGA in its 1000 bp upstream sequence (data not shown). Thus, the presence of more copies of GANNNNGA in the upstream sequences of miRNAs may signal a globally conserved function, such as tissue specific expression. We also found the frequency distribution of the motif in the upstream sequences from human and mouse orthologs of *mir-1 *and *mir-124 *to approximately follow its corresponding distributions in the two worms, thus confirming the conserved nature of the motif.

Motifs CTCTCTCTCTC and CTTCTTCTTCTTC, complementary to the motif GANNNNGA were found earlier in the studies of *C. elegans *muscle specific regulatory motifs [26]. We found these motifs also from the *mir-1 *and *mir-124 *1000 bp upstream sequences in *C. elegans *and *C. briggsae*, thus strengthening the connection of these miRNAs with a muscle specific function in these two worms.

Computational identification of miRNA genes relies upon the properties of known miRNAs such as the length of mature miRNA and their hairpin precursor secondary structure, together with high phylogenetic conservation of some miRNAs [18,27,28]. The predictions based on the miRNA hairpin sequence only are not very accurate and tend to give a high number of false positives. Computational algorithms are also prone to false negatives and may miss miRNAs that do not have clear homologs in related species. The accuracy of the algorithms can be improved by adding characteristics from miRNA flanking sequence, such as scoring the sequence conservation and characteristic motifs [11]. Ohler and his co-workers successfully applied knowledge about the motif CTCCGCCC and its location upstream from the orthologous independently transcribed miRNA foldbacks to improve the accuracy of the miRNA gene prediction. In our study, this motif was found also from upstream of 10 *C. elegans *miRNAs located in the sense strand of an intron (data not shown), thus suggesting that it could be used also to improve prediction of the intronic miRNAs. In addition, the motif GANNNNGA is highly conserved in the vicinity of the miRNA starting site and equally frequent in the upstream of all the miRNAs despite their genomic location, suggesting that it could be a useful additional characteristic for the search and computational prediction of miRNA sequences.

## Conclusion

To better understand the transcriptional regulation of miRNAs, we searched for conserved sequence motifs from the area 1000 bp upstream from all miRNAs independent of their genomic location. We discovered one significant sequence motif GANNNNGA, occurring in every miRNA upstream sequence and having a conserved distribution in the upstream sequences of all miRNAs of nematode worms *C. elegans *and *C. briggsae*. It appears to be a common factor for the upstream regions of all miRNAs and is concentrated in a proximal area near the beginning of the miRNA precursor start sites. It may not be a transcription factor binding site, but rather a co-factor site, or recognition sequence for miRNA processing. The abundance and conservation of this motif in the upstream of two old and important miRNAs, *mir-1 *and *mir-124 *suggest a connection to miRNAs with global specialized function. The abundance and location of the GANNNNGA motif may be of benefit in computational prediction of miRNAs.

## Methods

### Datasets

We downloaded from WormBase release WS160, 31 July 2006 [29] the sequences 1000 bp upstream from the beginning of all available *C. elegans *and *C. briggsae *miRNAs. We considered as clustered those *C. elegans *miRNAs that were less than 1000 bp distance from each other. Seven clusters containing 23 miRNAs in total were found and only the upstream sequence of one, upstream-most miRNA from each cluster, was chosen to represent the whole cluster. Thus, the number of *C. elegans *1000 bp miRNA upstream sequences was reduced from 116 to 100 while for *C. briggsae*, there were 95 miRNAs in all. In this study, these two datasets were considered as putative miRNA regulatory sequence sets. From the 100 *C. elegans *miRNAs included into this study, 75 are located into the noncoding area of the genome and 25 are located in the gene coding area. We selected the 1000 bp upstream from intergenic miRNAs, rather than from their host genes, in order to find regulatory motifs specific for miRNA sequences.

### Motif extraction

Within both of our data sets we searched for short, over-represented patterns to find putative regulation motifs. For this purpose we used four motif finding tools available: POCO, Weeder, MEME and Gibbs Motif Sampler. POCO [19], takes advantage of exhaustive search methods and bootstrapping to find significant patterns composed of the nucleotides A, C, G, T and N from a given data set compared with the selected background organism. The resulting patterns contain at least 4 known nucleotides in steady places while the first or the last nucleotide must not be the wildcard N. Thus, mismatches are allowed only in fixed places in the pattern. All the full-length patterns and their sub-patterns are automatically analyzed, so when the maximum possible length of searched patterns, 8 bp, is selected for pattern length, the output contains all significant patterns which are 4–8 bp long. We used POCO in our miRNA upstream sequence motif analysis because this kind of motif model has not been used earlier in similar studies [7,11-13], and so it could give a new perspective to the possible regulative elements of miRNA genes. For the background organism to be used in the analysis we chose *C. elegans clean *which contains the promoter regions (downloaded from Ensmart [30], parameters: known genes, one output per gene, 5' upstream only and 5' Flank 3000 bp [19]) of almost every gene in the *C. elegans *genome where sequences containing letters other than A, C, G or T are removed. We chose the maximum 8 for pattern length and left all the remaining parameters as default. Weeder [31,32] is an exhaustive enumeration method for finding approximate occurrences of patterns. Significance of the found motif is calculated against the oligo frequency in the protein coding gene upstream regions of the selected organism. We chose to use Weeder in this study, because it has proved to perform well when compared to other common motif finding tools [33]. We ran Weeder with "quick" mode, and looked for motifs of length 8 allowing 2 mutations. MEME [34] is a probabilistic local alignment based method for discovering degenerate sequence motifs. The significance of the found motif is calculated against random sequences with the same nucleotide composition as the query sequences, so the motifs E-value refer to the expected number of motifs of equal width with the same or higher likelihood in a random sequence set with the same size and nucleotide composition as the input sequence set. We used MEME because it is used also in the other miRNA upstream motif studies [7,11,13], which gives a possibility to compare the results. In this study, MEME was launched to search motifs with width 8 bp having any number of repetitions in the input sequence set. The optional attributes were left as default. The Gibbs Motif Sampler is a probabilistic, alignment based approach for locating common motifs in collections of sequences [35]. The Gibbs Recursive Sampler [36] is a version of Gibbs sampler, developed for locating multiple transcription factor binding sites simultaneously from unaligned heterogenous DNA sequences. In this study, we launched the Gibbs Recursive Sampler with the minimum attributes: the number of different motifs was set to 3 and for each of them the initial width estimate was set to 8, and the maximum number of sites per sequence was set to 10. Even though the initial width of the sites was entered as 8, the output sites had length of 8 or more, where 8 columns were conserved and the rest of them were non-conserved.

### Motif analysis

From POCO results for both worms, we chose such patterns that were found from every sequence in the putative miRNA regulatory sequence sets, having *p*-values less than 0.05 and searched among them for patterns common to both sets (for algorithm, see Additional file 3. These patterns were then aligned with ClustalW [37], (Additional file 4).

The most significant motifs were located from the putative miRNA regulatory region with Visualize tool of POXO tool series [38], or Weeder Motif Locator included in MoD Tools [32], allowing two mismatches to the consensus sequence, and the position weight matrices were calculated from these results. The motif logos were created with WebLogo [39] and Transfac and Jaspar databases were searched for matching TFBSs with STAMP [21].

To test the statistical significances of the found motifs, we generated 100 random 1000 bp long sequences with the same nucleotide composition as *C. elegans *genome and counted the motif occurrences from these sequences. Then we calculated the *p*-values for the putative motifs with the independent samples t-test.

The 1000 bp upstream sequences of human and mouse miRNAs that belong to *mir-1 *and *mir-124 *families were downloaded from UCSC Genome Browser [40], and the multiple sequence alignments for the *mir-1 *and *mir-124 *families upstream sequences were made with ClustalW [37].

Additional file 5 shows the detailed workflow of the discovery and study of motif GANNNNGA.

### miRNA microarray analysis

Wild type worms (strain N2) were fed with OP50 *E. coli *on culturing plates containing Nematode Growth Media (NGM) agar [41]. Animals were grown at 20°C, synchronized and harvested at Larva 4 (L4) stage. mirVanaTM miRNA Isolation-protocol (Ambion Inc., Austin, Texas) was used to isolate small (<200 nucleotide) RNAs containing miRNAs which were labeled using NCodeTM miRNA Labeling System (Invitrogen, Carlsbad, CA) using the manufacturer's protocol. Labelled RNA was hybridized on NCodeTM Multi-Species miRNA Microarray V2-arrays (Invitrogen) containing three sub arrays (technical replicates) with 115 probes for *C. elegans *miRNAs. Arrays were scanned using ScanArray 5000 (GSI Lumonics, Billerica, MA) and images were converted as numerical form using TIGR Spotfinder- software [42] resulting in raw signal intensity values. Mean signal value for each miRNA was calculated from seven independently hybridized arrays.

## Authors' contributions

LH planned and conducted the experiments, and wrote the manuscript. SA conducted experiments and contributed to writing the manuscript. GW conceived the study, planned the experiments, and wrote the manuscript.

## Supplementary Material

Additional file 1 **The multiple sequence alignment of the *mir-1 *family upstream sequences of *C. elegans*, *C. briggsae*, human and mouse.** Click here for file

Additional file 2 **The multiple sequence alignment of the *mir-124 *family upstream sequences of *C. elegans*, *C. briggsae*, human and mouse.** Click here for file

Additional file 3 **The algorithm applied to find common patterns from two sets.** Click here for file

Additional file 4 **The multiple sequence alignment of conserved patterns found with POCO.** Click here for file

Additional file 5 **The detailed workflow of the discovery and study of motif GANNNNGA.** Click here for file
