# Supplementary material for: Identification of phylogenetically conserved sequence motifs in microRNA 5' flanking sites from C. elegans and C. briggsae
Source: BMC Mol Biol. 2008 Nov 26;9:105. doi: 10.1186/1471-2199-9-105 (PMC2613404; doi:10.1186/1471-2199-9-105)
Supplement: Additional file 1 — The multiple sequence alignment of the mir-1 family upstream sequences of C. elegans, C. briggsae, human and mouse. [file 1471-2199-9-105-S1.doc]

### Supplementary Figure 4 – The multiple sequence alignment of the *mir-1* family upstream sequences of *C.elegans*, *C.briggsae*, human and mouse.

cel-mir-1 TGAGCATTAAAAGTTTATGAACCCTAGCGAG--ATTGTTTAGAAATACTAAATATTGCTC 58

cbr-mir-1 -----------AGC**TCTTCATC**C---AATAG--AATGCCT**TCCAATTC**CG--TTTTTATC 42

hsa-mir-1-1 --GGGTGGTGACGCTGCGAGGCGGGGACGGGGGA**GAGGGTGAAGAAGAGCGAGAGAAG-G** 57

mmu-mir-1-1 --CCTTTGTGTAGCTACAAA-CTACTATTGATCAGGGACTGATG**GATCAGGA**ACTAGGTG 57

* * * * * *

cel-mir-1 ACAAATAGCAATTTTTCAAAAATTAATTTTATTCCTCTTTACCATA--TCCAATTAATTC 116

cbr-mir-1 A-AGATATCA-TGTTTCAG----TACCTATCTTCTTAATTACCAAGAGTTGACCCTATTC 96

hsa-mir-1-1 AGGGAGAGAACCGGGGGAG-----CGACGCTCCCTCGCCCACTGGGTGTCTGCTGTGGCC 112

mmu-mir-1-1 AGGGACAGGGCCCTTC-AG-----AAATAGCCTCCAGATTTCCATGGGAGCGGTTCCTTA 111

* * * * * *

cel-mir-1 TTTGGAGATCTTAATTACAAAA**GACCCTGA**CCTA**TCTCTTTC**ACCCATATCAGATTTCAT 176

cbr-mir-1 TT**TCTTCCTC**CCA--CACTAGCGATCGCTTCTCAGA--TTTCACCT---CCAGTGTCGAT 149

hsa-mir-1-1 AGGCTGAGGCACC--TAGGGAGTTGCCCCCCTAGTGGACGGCCTCT----CAGGA--**GAT** 164

mmu-mir-1-1 CGACCATCTTACA--TGGTTATGCTGCATAGCAGGGGATGGCGTT-------GGA--GAC 160

* * *

cel-mir-1 TGAAATTATTACAGTAGTCTTTGA---ACATCTGTGACTC**TCAAACTC**TTATAATCTCTT 233

cbr-mir-1 TTAAATTATTACAGTAATCCCCCAGAGATG**TCTGAATC**TCTTCGACTC**TCAAACTC**TCTA 209

hsa-mir-1-1 **CCTGA**CCACCCTCTGGGAACCTGGACTCCTCCAAGCATCG**TCTTTCTC**GCCTGCATCTTC 224

mmu-mir-1-1 TGTAGGT-CACCAGGAGGACAGAGGGTAC----AGAAGCGAGAAGAGG**GAAGGGGA**CCAA 215

cel-mir-1 ACAATTA-CTTGTTTCGCCTGG--C**TCCAACTC**TACTGTACAACCAACTC-TCTTTTCCC 289

cbr-mir-1 ACGGTTAACTCGTTCCGTCTGGTGC**TCCAACTC**CACTGTACAACAACCCTA**TCCTCCTC**C 269

hsa-mir-1-1 GGAGAGAGGT-GCTGAGCC-----CTTCCTCATGGATACGGGGTC---CCGGGAGGCAGC 275

mmu-mir-1-1 AGAAGGAGGTAGCTGGAATAGAAACTCCCTCATTGGCTTGGCATCAACTCTCAGGCCAGA 275

* * * * ** * *

cel-mir-1 **TCCTCCTCATCCTACTC**AGA**GAGAAAGAGA**GAAATGGGAAAAGGTACGCCTC-------C 342

cbr-mir-1 **TCTTCCTC**ATTGCAAAGAGAAAAAAAGAAAGAAA-GACATAAGGTACGCCTC-------C 321

hsa-mir-1-1 CTT--CACCAGGGACCTGCCCAGGAAACCAGCAGATGCCCAGGTAAGCCCCCAGGACACC 333

mmu-mir-1-1 CTGAGCCCCAAAGAGCTGGCCAAGGAGTAGGTCACCTCTTAG--TAGCCCCTAGA----C 329

* * * * * * * * ** *

cel-mir-1 TTCCAAAAAAT--AGGCGGAGTCTGGG------AAGGACAAGAAGAAGCAGCGAGAAGCC 394

cbr-mir-1 TTCCAAAAAAACGGGGCGGAGTCTTGGGACTAGAAGCAAGGGAAGAAGG**GACGGGGA**AAC 381

hsa-mir-1-1 TCTCGGGTCTCTGGTCTGCAGT**TCCTCGTC**T--GGCCTCAGGAGCAAGGAGTTG**TCCAGC** 391

mmu-mir-1-1 ACTGTGAAT**TCAAGATCACTATC**TT**TCTTCT--TC**ACTTCAAAGAAAGAGGCACTGAGAC 387

* * *** *

cel-mir-1 AGTC--CCTACACCCAAACCAACC---GAAAGACA-----TTCTTCCGA**TCATTCTC**GCT 444

cbr-mir-1 AGCCAG**TCCCCATC**AAAACCAACCCCC**GAAAGAGA**CGCATTTCTTCCGA**TCACCCTC**TCT 441

hsa-mir-1-1 **TC**CCG**GAGAAGGA**GGAGGCTGGTCCT-GCGAGTGGGGTGCTGGGCAGGGCGCCCCCCAAG 450

mmu-mir-1-1 CTTCTCTC---GGGGACTCTCATTTT-GGGAAACAA---TTCA**TCAGGGTC**CTGCCAGGG 440

* * * * * * * *

cel-mir-1 **CTCTCTAGTTCTCTTCCTCGTTTTCTT--TCTCTCTTCT-TC**TTTTTAAATATCGCCA-A 500

cbr-mir-1 TTCCTTATG**TCTCTCTCCCTTTC**TTTC--TTTAAAATATATCGTGCCGTCCGTCCCTATG 499

hsa-mir-1-1 GTTCCTCTCTCCACCCAGCCCTCTCTGGGCTTGGCTGGGATGAGGTGACCCAGGGGGACA 510

mmu-mir-1-1 GAGCCATCGCCTGTCCAGGTAAGCCC---TTTGATTCATTTCTGGAGACTCACA------ 491

* * *

cel-mir-1 TTTTTCCATT------CGATGCCTGTTAAGACGGCAATGACGGAGGAATGA---GTA**GAA** 551

cbr-mir-1 TTTTTCCCTCTTTTTGCAATGCCTGTTAA**GACGGCGA**TGACGGAGGAATGACAT**GAATAA** 559

hsa-mir-1-1 GGAAAGTGTTGGCTTTGAAAGTTTGG**GACATGGA**TGATGCCTCCCTGCTCTGGGGTGAGG 570

mmu-mir-1-1 --AAACTGCTTGTTTTCA---TCTG---TCCTCAGGGAGCTGGGCTGTTCAAGTCCCCAG 543

** * *

cel-mir-1 **AAAGACGAAGAAGA**GAGGCC---TCTCTGTACG----TGTGCAAATATATTTGCTCAGTG 604

cbr-mir-1 **GAAGAAGAGGAAGAGAGACCGA**ATTTGCCTGTG----TGTGCACATATATTTGCTCAGTG 615

hsa-mir-1-1 GTGGAGGGGCAGCCTGTCTCTTCCATGGACGCAAAACCGAGCTACTGCTGGGGCCAGGGA 630

mmu-mir-1-1 **GAAAAGGA**TGTGACTGGT**TCTTAGTC**TGGGGTA----CGGGTCCCTGATGGCTCGTGGGT 599

* * * * * * * *

cel-mir-1 CGCACGCACCTTCTTTTCTTAGGTCGAACC--CCACCCATCCTCTCTACAAATCTCCCGC 662

cbr-mir-1 CGCAC----CTTTTCTTCTTAGGTCAACCC--CACCCCATCCCTTACAAA**TCTCTCTC**GT 669

hsa-mir-1-1 CAGGC-GCTCGAGACTTTCTGGGGC**TCACTGTCCACTTC**TGCCTT**TCTGGATC**GTGTGA- 688

mmu-mir-1-1 GGAGCTGCTCCTGAAT**TCTGTCTC**CTTACC--CCACCC**TCACTGTC**CTTGGTGGTGACAA 657

* * ** * * * * * * *

cel-mir-1 TAC-----CTTCATAC-----------------AAACACAC------TG-TAGACTTACT 693

cbr-mir-1 TACTACACCTGAAGACTGTAA-----------GAAGCATTC------TGATAGGCATAGT 712

hsa-mir-1-1 -GTGTGT**GAGACAGACAGAGA**-----------**GAGATGGA**T--------TCAGGGATGGA 728

mmu-mir-1-1 GGTGGGCAG**GAAGGAGAGAGA**AGATGTTAGTA**GAGTTGGA**TGCTATTTCTTAGAGTCTGG 717

* * **

cel-mir-1 -TGGT**TCAAGTTC**CAAGTTACTATTGG--GTTTAGTCTTACGTTCATTTGTGTAGTAGTC 750

cbr-mir-1 GTTTT**TCGACGTC**TTCCGTATTCCAAA--AAATCGAAACCAGACTGGCAAGATAGCTCGC 770

hsa-mir-1-1 GGGGCG-----CCGAGGTCAGAAGGGGGCAGGGAGTCCTGGGCCCCTGGGGGTGGCTGGA 783

mmu-mir-1-1 GATGTGACTTCCCTCTGTTGGGAA**GAATGGGA**AAGAACTCATTCCGTTGAGGGAGGTGTA 777

* * *

cel-mir-1 ACTTACCATACTAAG--TTGATTGGAGAAACTTATTGTAGAGAAGAGAGTGTAAGCTGAT 808

cbr-mir-1 TCTGAACCTAGGGAAGCTTGAACTGTATTCTTTTTTCCCCACAAAAATTTGTTCTTTGGT 830

hsa-mir-1-1 CACCA-GGCAGCAGTGGCAGGAAGGCTG**T--CCTGCTC**ACACAGAGAGGGCTCCGGCAGT 840

mmu-mir-1-1 AGCAACGGTGGGGATGAAAAGGGTTTTGAGACTTTTCAGCACGCCCTG**TCTGCTTC**CAGT 837

* * *

cel-mir-1 TTAAACTTCTCTTCAACTCTCCGAAGAATTTTTTGATCTCCCAAATCTTTCAC-GAGTCG 867

cbr-mir-1 TGTCTTAAAATCTGAGATTCCAGAAACGACTTCCAAATTTTAAACTTCTTCACTGACTTC 890

hsa-mir-1-1 AGACTCCAGGGAAGAAGTTAC-ACTGCCTCTGA-GCTGCCTTC**TCTACATC**GCAGTGGGG 898

mmu-mir-1-1 CTTTACCAAGTGTGCATGTGT**GAGAGAGACTGA-G--A**CACAGGCGACACCAAAAGGGGG 894

* *

cel-mir-1 T---CTTTTCCCAGATTCAATTC-TAC**TCTATATC**TACGCTATATTCAAAGTG---ACCG 920

cbr-mir-1 TATACCGTTC**TCTGATTC**ATCTGATATTCTGCTATTATTCCAACCAAAAAACCCCTGCTG 950

hsa-mir-1-1 **TCAGCTTC**TACCGGGGCGGCGTCCCGGGGTCTTGGAACTGCATGCA----GAC--TGCCT 952

mmu-mir-1-1 CCAGCTTGTGGAGGGCCACTTCC**TCGGAGTCCTGTTC**TTGTGCACACTGAACC--TACCT 952

* * * * *

cel-mir-1 TACCGAGCTGCATACTTCCTTACATGCCCATACTATATCATAAATG**GATATGGA**ATGTAA 980

cbr-mir-1 TGCCGAGCTGCATACTTCCTTACATGCCCATACTGTACTGTGAATGGATA---------- 1000

hsa-mir-1-1 GCTTGGGAAACATACTTCTTTATATGCCCATATGGACCTGCTAAGCTA------------ 1000

mmu-mir-1-1 GCTTGGGACACATACTTCTTTATATGCCCATATGAACCTGCTAAGCTA------------ 1000

* * ******** *** ********* ** *

cel-mir-1 AGAAGTATGTAGAACGGGGT 1000

cbr-mir-1 --------------------

hsa-mir-1-1 --------------------

mmu-mir-1-1 --------------------
