# Supplementary material for: Identification of phylogenetically conserved sequence motifs in microRNA 5' flanking sites from C. elegans and C. briggsae
Source: BMC Mol Biol. 2008 Nov 26;9:105. doi: 10.1186/1471-2199-9-105 (PMC2613404; doi:10.1186/1471-2199-9-105)
Supplement: Additional file 2 — The multiple sequence alignment of the mir-124 family upstream sequences of C. elegans, C. briggsae, human and mouse. [file 1471-2199-9-105-S2.doc]

### Supplementary Figure 5 – The multiple sequence alignment of the *mir-124* family upstream sequences of *C.elegans*, *C.briggsae*, human and mouse.

hsa-mir124a-1 CGCCTCTCCTCCCTCCCTCCCCCCTGCACTCCTCTC**TCTTCCTC**CCTCCCACCTCCCTCG 60

mmu-mir-124a-1 ------------------------------------------------------------

cel-mir-124 ------------------------------------------------------------

cbr-mir-124 ------------------------------------------------------------

hsa-mir124a-1 CTCCCGCCCTCCCTGCTGCATTTTGCATGAGCTA**TCTAGGTC**ATTAGCATTTTAGCGTAT 120

mmu-mir-124a-1 -----------------------------AGCTA**TCTAGGTC**ATTAGCATTTTAGCGTAT 31

cel-mir-124 --------------------------------------------GTGCACTTGATTCTGG 16

cbr-mir-124 ----------ATCACACCTGCTTCTTAGGCAA**TCCTTTTC**TAATGAACGTTTAATTATAG 50

* ** * *

hsa-mir124a-1 GCAAGACTTGACAAAGCTGATGAGGGGCCAGATGGGCCTCGCTCTTATGGTTTTTTTTTC 180

mmu-mir-124a-1 GCAAGACTTGACAAGGCTGATGAGGGGCCAGATGGGCCTCGCTCTTATTTATTTATTTTT 91

cel-mir-124 A--CCCATCGA**GAAAGTGA**ACATGTGCTTTAACGAAAT-ACATCACATT-------TTCT 66

cbr-mir-124 GATCTCAGTACTTCAGTGC-CAGATTGTTTACTGAAATGACAG**GAAATTGA**GAAAATAAT 109

* * ** *

hsa-mir124a-1 T**TCTTCTTCTTCTTCTTCCTCTCTCTCTCTCTCTCTCTGTCTCTCTCTCTCTC**GTCTCTC 240

mmu-mir-124a-1 TTTTTCTT---------------------------------------------------- 99

cel-mir-124 CTT**TCCTATTC**ATG---------------------------------------------- 80

cbr-mir-124 TTTGTGG**GAGCCTGA**AA------------------------------------------- 126

*

hsa-mir124a-1 TTCCTTTCCCCCCTCCACCACTTGGGACCT**GAGCGAGA**GGACTGC-AGCAGGCGAGTTCC 299

mmu-mir-124a-1 TTCCTTTTTCCCATCCACCACTTGGGACCTAAGCGAGAGGACCGC-AGCAGGAGAGTTCC 158

cel-mir-124 -----TGCCTACAATAACAAAGTTGCACCTACTCTGTAGGTATGCAGGTACGCACACCAT 135

cbr-mir-124 GTTATCGTCAACAACAGCAACGTGGCT**TCTGAATC**AACTGCTTATCAGTATTCAAGAGAA 186

* * * * * ** * * *

hsa-mir124a-1 GGAAGGCTGAATCCC-CAGGTCCCAGCCCCGGACCGCGGCAACTCGCCCCTGCGGCACGC 358

mmu-mir-124a-1 GGAAGGCTGATTCCCACAGATCCTGGTAAGGGACCGCAGCAGCTCTGCCCTGCGGCACGC 218

cel-mir-124 TGAAAAAAGGT---TTTTGGATGCT-TCCATAAT----GTAATTAACTTTTAAAGAATTC 187

cbr-mir-124 AGGTGTGAGATAAGC**TCTGAATC**CCATATCCAAC----ACAATTTAATCCAATCCGTTTT 242

* * * * * *

hsa-mir124a-1 CCGGCC-CGGCTGCGCCCCCACTCAGCGATGGAGGTAAAGAGGCGGCGGCCGCGCGCCCT 417

mmu-mir-124a-1 CCGGCC-AGGCTACACCCGGACCCTACACTCAAGGTAAAG--GCGGCGGTCACAAGCCCT 275

cel-mir-124 ACTTTTGCGTCTGTTGTCACACATGACAAAG**TCCCTATC**T---CCCCTTCCATGCTCCAG 244

cbr-mir-124 C**TCGTTTTC**TCTGATATCATATATC-CAGCGGAAACGT-T---TCCTGGCAACGCTACAT 297

** * * * *

hsa-mir124a-1 GCCCCGGCTGCCGCTGCCGAGTCCCCGCTGCTC**TCCTTGTC**CT**TCGCTCTCTTCTTCCTC** 477

mmu-mir-124a-1 GCCCAGGCAGCAGTAGCTGGGCCCCTGCTTCCCA**TCCCTTTC**C**TCTCCGTC**A**TCTCCCTC** 335

cel-mir-124 ACAGCAACCACCAGTTGATAACATTTCAATACTTTTCTTTTCTAT-CTGTTACTTTTATC 303

cbr-mir-124 GTGATAAGCCCAAATTCT---CACTTCTTTTCTTCCTATTTCGATTC**TCTTGCTC**TCATG 354

* * * * * *

hsa-mir124a-1 C**TCCTAGTCCCCTTCAGTTTC**CTGGGCGAA-GCA**GAGGGCGA**CATGGGTGGGTGGG--TT 534

mmu-mir-124a-1 CTCCTGGCCCCCC**TCAGTTTC**CTAGGCGAA-GCT**GAGAGCGA**ACTGGGTGGGTAGGATTC 394

cel-mir-124 CTCCA-CTGTTTTTATGCTCTCTGAGCATCTACA**GAAGTAGA**ACTTGGCGCCGTGCGTCT 362

cbr-mir-124 CTCT--C**TCTCTCTC**T-CTCTCTATGCATCTACAAAAG-ATAACTTGGCGCCACCAGTCC 410

*** * * ** ** * * * * ** *

hsa-mir124a-1 GCTGCGCTGGGGCGAGGTGGGGTCGATGT-TGTTTTT**TCATTGTC**TGGAGCTGCAGGGGA 593

mmu-mir-124a-1 GCTGCGGTGGGGCGAGGTGGGGTTGATGT-T---TTT**TCATTGTC**TGGAACTGCAGGGGA 450

cel-mir-124 ATCGATTTTCGTTGACGGGCCCCCGCGGTCTATCTGCGCAATTC**TCGAAGTCG-AGTC**TC 421

cbr-mir-124 ATCGATTTTCGACGACGG---CAAGAGGTCTATCCGCGCAATTCTCGACGGTGCAGTCTC 467

* * * ** * * ** * ** * * * **

hsa-mir124a-1 GGCGAGGCGCGGGGAAAGGGGCGAGGGGAGCCGGGGTAATTAACACGGGGGAGGCACC-- 651

mmu-mir-124a-1 GGCGAAGCGCGAGGAGAGGAGTGAGGG-AGTCGGGGTAATTAACACGGGGGAGGCACC-- 507

cel-mir-124 CACAGATTCAATACTATCTCATCTCGGTGCTCACGTCGTCTAGCAC---GAAGCCGTC-- 476

cbr-mir-124 TTTAGATTCAACACTATCTCATCA-GCAGCTCACGTCGTCTAAAAGAGAGAAGCCGTCGT 526

* * * ** * * ** * *

hsa-mir124a-1 --CC**TCCGTCTC**CCACTT-CCACCCACACCCCCATC----------------------CC 686

mmu-mir-124a-1 --CC**TCCGTCTC**CCACTT-CCACCCACATCCCGAAC----------------------CC 542

cel-mir-124 ---CTCCGCCTTCCCCTTTCCTTTT**TCGTTCTC**TTC--------------------TTTC 513

cbr-mir-124 C**TCCTCCTCCTCCTCCTTCTC**TTGTCCG**TCTTCTTCGCTTTC**CT**TCCATGTCTCTTTTTC** 586

**** ** * *** * * * *

hsa-mir124a-1 TCCACCCCC-TCCGCTTTGCAGGAAAAAGCCTGGATGCGAAAGGATGGGGGAGAACAAAG 745

mmu-mir-124a-1 TCCACCCCCCTCCCCTTTGCAGGAAAAAGCTTGGAAACGAAAGGATGCGGGAGAACAAAG 602

cel-mir-124 TTCTT**--**T**C**T**TCTCATTCGCAGTC**CGTTGCTGT---CGCCGACGCCGCCGCGGCTCTTGA 568

cbr-mir-124 T**TCTTCTTCTTCTCATTCGCAGTC**CTTCGCCGTG**GACGACGA**CGTCGCCGCGGCTCTTGA 646

* * * ** ** **** ** * * * * * *

hsa-mir124a-1 AGCCTTTGGAAGACGTCGCTGTTA---TC**TCATTGTC**TGTGTGATTGGGGGAGCTGCGGC 802

mmu-mir-124a-1 AGCCTACGGAGGACGTGGCTGTTA---TC**TCATTGTC**TGTG--ACTGGGGGAGCTGCGGT 657

cel-mir-124 CTAGTAACCCGCTCTCTTCTACCAAACAACCAGTATTCATGCTTTGCT**TCTTCTTC**GAGC 628

cbr-mir-124 CTAGTAACC--CCCACCACCACC--ACAACTAGTATTCGTGCTTT---------TCGAGC 693

* * * * * * ** * *

hsa-mir124a-1 GGGGAGGATGCTGTGGTCCCTTCCTC-CGGCGTTCCCCACCCCCATCCCT-CTCCCCGCT 860

mmu-mir-124a-1 AGGAAGGATGCGGTGGTCCTTAGCTCA**TCGCGTTC**CCCAACCCCACACC--CTCCCTGCT 715

cel-mir-124 ACACAGGAGGCATGTAATC--TCTCCGGTGAACCACCTGCCTTGGCTTCCCCTAGGCACC 686

cbr-mir-124 ACATAG----CATGTAATCCTTTTTCGGTGAACCACCTGCCCTTGTCCG--------ACC 741

** * * * * ** * *

hsa-mir124a-1 GTCAGTGCGCACGCACACGCGCC-----------------GCTTTTTATTTCTT------ 897

mmu-mir-124a-1 GTCAGTGCGCACGCACACGCACGCG---------TCGCCAGCTTTT**TCTTTCTC**A----- 761

cel-mir-124 ATGGCAGTCTAATCCA**GAGCGAGAGAGAGAGA**AAAGACCTCC**TCCTTCTCTCTCTCTCTC** 746

cbr-mir-124 ATAAGCATCTAATCCAGAG---------------AGACCTCCTTCTTCCCTAT------- 779

* * * * ** ** * *

hsa-mir124a-1 --TTTCCTGGTTT**TCTTATTC**CATCTTCTACCCACCCCTCTTCCTT--TCTTTCAC---- 949

mmu-mir-124a-1 --TCTCTCATTTTTTTCATC**TCATCTTCTCCCCATC**CCC**TCCCTTT**--**C**TTTCCAC---- 813

cel-mir-124 TCTCTACCACCTGCCCAAATGAGAAATGACTCCGCCCTCGTCGCTCATTGATTCACATCC 806

cbr-mir-124 ----GACCACCTGCCTAAA-AAGAAAGAAGT--GGCCACGCCCCTC---GATTCAC---- 825

* * ** * * ***

hsa-mir124a-1 --CTTTCCTTCCTTCCTTCCTCCT-TTCCTTCCTCAGGAGAAAGGCCTCTCTCT------ 1000

mmu-mir-124a-1 --CTTTCCTTCCTT**TCTTCCTCCC-TTC**CTTTCTCCCATCC**TCCCTCTC**TTCCATCCTTC 870

cel-mir-124 ATTTT**GAGAAGGA**TGGTTGCTACTGCTGCTTGATCTCAAGGACTGTGTGCTC--CCACTT 864

cbr-mir-124 ----TGGACACATTCATTG-TGC--CT**TCTTGATC**TGAAGGACTGTC**TCTTCTTC**TCCTA 878

* * ** * * * *** ** *

hsa-mir124a-1 ------------------------------------------------------------

mmu-mir-124a-1 CTTCCTTTCCATCCTTCCTTCTTTCTTTC---CTTCCTTCCTTCTTTCTTTCCTTCCTTC 927

cel-mir-124 TTACTCAAAGTATTTTATT**TCTTCTTC**AAGTGCTCTCTTCTCCATTTTTGAATTTCCTTC 924

cbr-mir-124 TTACCCCAG--AGCTAATTTCATTCTTGA-----------CCTACTCAAGTGCTTCTTAT 925

hsa-mir124a-1 ------------------------------------------------------------

mmu-mir-124a-1 CTTCTTCCTTCCTCAGGAGAAAGGCC**TCTCTCTC**CGTGTTCACAGCG**GACCTTGA**TTTAA 987

cel-mir-124 GCAATTGATACACCATGTCCCACTTGTCATCTGGCATGCACCCTAGTGACTTTAGTGGAC 984

cbr-mir-124 GTCATTGATACATTTT-TTCCAGTCGTCATATGGCGTCCACCTGAGTGACTTTAGTGGAC 984

hsa-mir124a-1 ----------------

mmu-mir-124a-1 ATGTCCATACAAT--- 1000

cel-mir-124 A**TCTAAGTC**TTCCAAC 1000

cbr-mir-124 ATGTATAGTTTCCAAC 1000
