# Supplementary material for: Identification of phylogenetically conserved sequence motifs in microRNA 5' flanking sites from C. elegans and C. briggsae
Source: BMC Mol Biol. 2008 Nov 26;9:105. doi: 10.1186/1471-2199-9-105 (PMC2613404; doi:10.1186/1471-2199-9-105)
Supplement: Additional file 3 — The algorithm applied to find common patterns from two sets. [file 1471-2199-9-105-S3.doc]

### Supplementary Figure 2 – The algorithm applied to find common patterns from two sets.
