# Supplementary material for: Identification of phylogenetically conserved sequence motifs in microRNA 5' flanking sites from C. elegans and C. briggsae
Source: BMC Mol Biol. 2008 Nov 26;9:105. doi: 10.1186/1471-2199-9-105 (PMC2613404; doi:10.1186/1471-2199-9-105)
Supplement: Additional file 4 — The multiple sequence alignment of conserved patterns found with POCO. [file 1471-2199-9-105-S4.doc]

### Supplementary Figure 3 – The multiple sequence alignment of conserved patterns found with POCO. The number of sequence on the left corresponds to its order on the list in Table 1.

1 --GAGA---- 4

27 --GNGAG--- 5

22 --ANGAG--- 5

24 --AAGNG--- 5

52 --ANGNGNG- 7

47 --ANGNGG-- 6

65 --ANNNGGNC 8

32 -GNNGNGA-- 7

40 -GNNGNGNA- 8

36 -GANGNG--- 6

42 --GGNGG--- 5

53 --AGGNG--- 5

2 -GANNNNGA- 8

19 -GANGNNG-- 7

12 -ANNGNNGA- 8

21 -GNNGNNGA- 8

8 --ANGNNGA- 7

56 --ANGGNG-- 6

61 --ANGGNNNG 8

63 --ANGNNNCG 8

5 -ANNNGNAG- 8

51 -ANNNGGC-- 7

44 -ANNGGNNG- 8

48 --ANNGGNG- 7

57 ---AGNGG-- 5

3 -GANANNG-- 7

43 -GNNANNGG- 8

15 -ANANGNG-- 7

23 --ANANGG-- 6

4 --ANAGNG-- 6

34 --ANAGNNNC 8

31 -ANNGNGNG- 8

49 --ANNGNGG- 7

18 --ANNNAGNG 8

6 -GANNNAG-- 7

60 GGNNNNAG-- 8

26 -GNNNGAG-- 7

37 GNANNNGG-- 8

38 -GNANGNG-- 7

7 --AGNGNNNA 8

41 --AGNGNNC- 7

9 --AGNGNA-- 6

11 --AGNGA--- 5

17 --AGNGNG-- 6

39 GNAGNNNG-- 8

59 GTNGNNNG-- 8

13 ---GAANG-- 5

62 --CGANNG-- 6

10 ---GAGNG-- 5

25 ---GANNGNG 7

20 -GNGNGNG-- 7

28 AGNNNGNG-- 8

14 -AGNAG---- 5

30 -AGNANNG-- 7

16 -GNNNANGA- 8

46 -GNGNANG-- 7

45 -GNGNNGG-- 7

29 -GNNGAG--- 6

33 AGNNNAG--- 7

35 GGNNGA---- 6

50 GGNNNANG-- 8

55 GNGNNANG-- 8

58 -GGNNAG--- 6

64 -GGNNNGG-- 7

54 -GGNNNNGG- 8
