# Supplementary material for: Identification of phylogenetically conserved sequence motifs in microRNA 5' flanking sites from C. elegans and C. briggsae
Source: BMC Mol Biol. 2008 Nov 26;9:105. doi: 10.1186/1471-2199-9-105 (PMC2613404; doi:10.1186/1471-2199-9-105)
Supplement: Additional file 5 — The detailed workflow of the discovery and study of motif GANNNNGA. [file 1471-2199-9-105-S5.doc]

### Supplementary Figure 1 – The detailed workflow of the discovery and study of motif GANNNNGA.

*Noticed similarity*

*Multiple sequence alignment*

*(MSA) with ClustalW*

*ClustalW*

*Comparison*

*Extract sequences 5000 bp upstream and 2000 bp downstream from each miRNA start site in C.elegans and C.briggsae*

*Scan for over-represented short patterns with POCO. Pick up patterns common to the sets.*

*Extract miRNA 1000 bp upstream sequences of C.elegans and C.briggsae*

*Extract 1000 bp mir-1 and mir-124 upstream sequences for human and mouse.*

*Locate GANNNNGA (Visualize).*

*Locate all occurrences of GANNNNGA in all the sequences (Visualize)*

Count and location of GANNNNGA in every miRNA upstream sequence of *C.elegans* and *C,briggsae*

Frequency distribution of GANNNNGA in the larger area around miRNAs in both worms

100 *C.elegans* miRNA upstream sequences

95 *C.briggsae* miRNA upstream sequences

Two sets containing longer sequences around the *C.elegans* and *C.briggsae* miRNAs

65 significant patterns common to *C.elegans* and *C.briggsae* miRNA upstream sequences

Consensus sequence GANNNNGNG

WormBase, miRBase

Count and location of GANNNNGA in the 7000 bp sequences around the

*C.elegans* and *C,briggsae* miRNAs

MSA of all mir-1, and for all mir-124 upstream sequences

Frequency diagrams of GANNNNGA for mir-1 and mir-124 in all four species

Count and location of GANNNNGA in

*mir-1* and *mir-124* upstream sequences of *C.elegans,* C*.briggsae,* human and mouse

*mir-1* and *mir-124* 1000 bp

upstream sequences of

*C.elegans,* C*.briggsae,*

human and mouse

The upstream sequences of *mir-1* and *mir-124* include high number of GANNNNGA in both worms

Focus on one pattern: GANNNNGA

miRBase, UCSC, ensEMBL
